# Using Mobile Phone Data to Estimate the Relationship between Population Flow and Influenza Infection Pathways

**DOI:** 10.3390/ijerph18147439

**Published:** 2021-07-12

**Authors:** Qiushi Chen, Michiko Tsubaki, Yasuhiro Minami, Kazutoshi Fujibayashi, Tetsuro Yumoto, Junzo Kamei, Yuka Yamada, Hidenori Kominato, Hideki Oono, Toshio Naito

**Affiliations:** 1 Graduate School of Informatics and Engineering, The University of Electro-Communications, 1-5-1 Chofugaoka, Chofu, Tokyo 182-8585, Japan; c2030076@edu.cc.uec.ac.jp (Q.C.); tsubaki@uec.ac.jp (M.T.); minami.yasuhiro@is.uec.ac.jp (Y.M.); 2 Department of General Medicine, Juntendo University Faculty of Medicine, 3-1-3 Hongo, Bunkyo-Ku, Tokyo 113-8421, Japan; naito@juntendo.ac.jp; 3 Division of Pharmacy Professional Development and Research, Hoshi University, 2-4-41 Ebara, Shinagawa-Ku, Tokyo 142-8501, Japan; t-yumoto@hoshi.ac.jp (T.Y.); kamei@hoshi.ac.jp (J.K.); 4 I&H Corporation, 1-18, Oomasu, Ashiya, Hyogo 659-0066, Japan; yamada_y@i-h-inc.co.jp (Y.Y.); kominato_h@i-h-inc.co.jp (H.K.); oono_h@i-h-inc.co.jp (H.O.)

**Keywords:** influenza, human/epidemiology, disease outbreaks, machine learning, neural networks, computer, geographic information systems

## Abstract

This study aimed to analyze population flow using global positioning system (GPS) location data and evaluate influenza infection pathways by determining the relationship between population flow and the number of drugs sold at pharmacies. Neural collective graphical models (NCGMs; Iwata and Shimizu 2019) were applied for 25 cell areas, each measuring 10 × 10 km^2^, in Osaka, Kyoto, Nara, and Hyogo prefectures to estimate population flow. An NCGM uses a neural network to incorporate the spatiotemporal dependency issue and reduce the estimated parameters. The prescription peaks between several cells with high population flow showed a high correlation with a delay of one to two days or with a seven-day time-lag. It was observed that not much population flows from one cell to the outside area on weekdays. This observation may have been due to geographical features and undeveloped transportation networks. The number of prescriptions for anti-influenza drugs in that cell remained low during the observation period. The present results indicate that influenza did not spread to areas with undeveloped traffic networks, and the peak number of drug prescriptions arrived with a time lag of several days in areas with a high amount of area-to-area movement due to commuting.

## 1. Introduction

Global excess mortality due to seasonal influenza-associated respiratory illnesses has been estimated to be 4–8.8 per 100,000 individuals and is a major issue in public health [1]. Designing efficient containment strategies for highly contagious diseases like influenza has become a subject of considerable interest [2,3]. Previous reports have focused on national epidemics among countries [2,4,5], but few reports have investigated the relationship between population flow and epidemics on a regional level. People move in small areas by various means, including walking, driving, or riding. Observing the subtle movements of large numbers of individuals has historically been limited by the availability of data. Therefore, it has remained difficult to observe population flow and epidemics in detail in local areas.

We have long been interested in information from mobile phones and early monitoring of influenza infections and developed and tested self-reported influenza infection monitoring applications from mobile phones [6]. Mobile phones can provide us with useful information for epidemiology. With the recent spread of mobile phones and the accumulation of location information, one can now trace the fine movements of many individuals. Hence, infectious disease epidemic tracking and forecasting have been attempted using data based on mobile phone Global Positioning System (GPS) location information [7,8,9,10,11]. Research goals related to population flow and infectious disease transmission, such as tracking overall trends, identifying unknown individuals who may have been infected, and verifying models and concepts, differ. Varying effectiveness has been shown, but on the other hand, standard methods have not been established, and it is still necessary to use various data, try many methods, and accumulate knowledge.

The prescription status of anti-influenza drugs is known to reflect the presence of an influenza pandemic [12,13]. We obtained the prescription status of anti-influenza drugs from a dispensing pharmacy in the Kansai region of Japan, and the location data on the movement and location of people in the Kansai region from a telecommunications carrier, KDDI Co. (Tokyo, Japan) that already has a track record in using population flow [14,15]. Disease mapping studies that monitor the spread of infectious diseases based on population flow have been conducted on a daily timescale for intercity locations [11,16], whereas mobile location data can be examined for specific areas and brief timeframes, thereby facilitating the observation of fluctuations on an hourly scale. Observing from a detailed perspective by increasing the information granularity may provide us with new insights. The present study aimed to estimate from a more detailed perspective influenza spread by observing the relationship between population flow during peak commuting times and prescribing anti-influenza prescriptions in the neighborhood using a specific method [17].

## 2. Materials and Methods

### 2.1. Analysis Data

#### 2.1.1. Number of Anti-Influenza Drug Prescriptions at Pharmacies

The first set of analysis data was the daily number of anti-influenza drug prescriptions filled between 1 January 2013 and 31 March 2019 at 86 pharmacies (managed by I & H Corporation, Hyogo, Japan) located in the four Kansai prefectures of Osaka, Kyoto, Nara, and Hyogo. The data collected for analysis in this study were the number of prescriptions filled during the study period for the following anti-influenza drugs: oseltamivir phosphate, zanamivir hydrate, laninamivir octanoate hydrate, and baloxavir marboxil. These data were not collected specifically for analysis in the present study, and all data were retrospectively retrieved from an institutional database (I & H Corporation). The lag in prescription distributions during this time can be estimated by comparing each region, but each pharmacy has different business hours, so the effect of closed days on estimating influenza infection pathways needed to be considered.

For example, using a hypothetical dataset as shown in Table 1, one can predict that there are a large number of individuals infected by influenza during the period that the pharmacy is closed from the prescription numbers. The number of prescriptions filled on Sunday is zero because the pharmacy is closed on that day, so if analyses are conducted on these data, the results would indicate that there are no individuals infected with influenza in this region on Sundays. However, it is more important to interpret this as individuals who wanted to go to a pharmacy on Sunday could not, and they thus chose to go to a hospital on Monday, thereby increasing the number of prescriptions on Monday. Therefore, a Kalman smoother was used on the anti-influenza drug prescription number data prior to analysis to smooth out the data.

A Kalman smoother was used for fixed-space smoothing problems, which estimates a state vector $x_{t}$ based on all observation data $z_{t}=\left\{z_{0},z_{1}\ldots ,z_{t}\right\}$ in the interval [0, t].

The discrete time model of the number of anti-influenza drug prescriptions was defined as follows:

(1) $$\begin{array}{c} x_{t}=F_{t}x_{t-1}+w_{t}\\ w_{t}\sim Gaussian\left(0,Q\right) \end{array}$$

(2) $$\begin{array}{c} z_{t}=H_{t}x_{t}+v_{t}\\ v_{t}\sim Gaussian\left(0,R\right) \end{array}$$

Here, x is the state vector, the number of anti-influenza drug prescriptions to be estimated, and $z_{t}$ is the observed value. $w_{t}$ is the process noise with a mean value of 0 and a covariance of Q, and $v_{t}$ is the observed noise with a mean value of 0 and a covariance of *R*. $x_{t}$ is generated by the above-mentioned state equation and the below-described conditional equation.

(3) $$\;x_{t}={\left[s_{t},s_{t-1},s_{t-2},d_{t},d_{t-1},d_{t-2},d_{t-3},d_{t-4},d_{t-5}\right]}^{t}$$

(4) $$F_{t}=\left[\begin{array}{cccccccc} 2 & -1 & 0 & 0 & 0 & 0 & 0 & 0\\ 1 & 0 & 0 & 0 & 0 & 0 & 0 & 0\\ 0 & 0 & -1 & -1 & -1 & -1 & -1 & -1\\ 0 & 0 & 1 & 0 & 0 & 0 & 0 & 0\\ 0 & 0 & 0 & 1 & 0 & 0 & 0 & 0\\ 0 & 0 & 0 & 0 & 1 & 0 & 0 & 0\\ 0 & 0 & 0 & 0 & 0 & 1 & 0 & 0\\ 0 & 0 & 0 & 0 & 0 & 0 & 1 & 0 \end{array}\right]$$

(5) $$H_{t}=\left[1,0,1,0,0,0,0,0\right]$$

Row 1 of Matrix (4) is $s_{t}=s_{t-1}+\left(s_{t-1}-s_{t-2}\right)=s_{t-1}+\left(\Delta s_{t-1}\right),$ and thus is a smoothed value, whereas row 2 is an identity function.

Additionally, $d_{t}$ expresses the corrected components by the day of the week, and row 3 of Matrix (4) indicates that the sum of all these corrected components equals zero. Row 4 onwards are identity functions, and vector $H_{t}$ indicates that the observed value is the product of the corrected components and the smoothed components.

**Figure A1:**
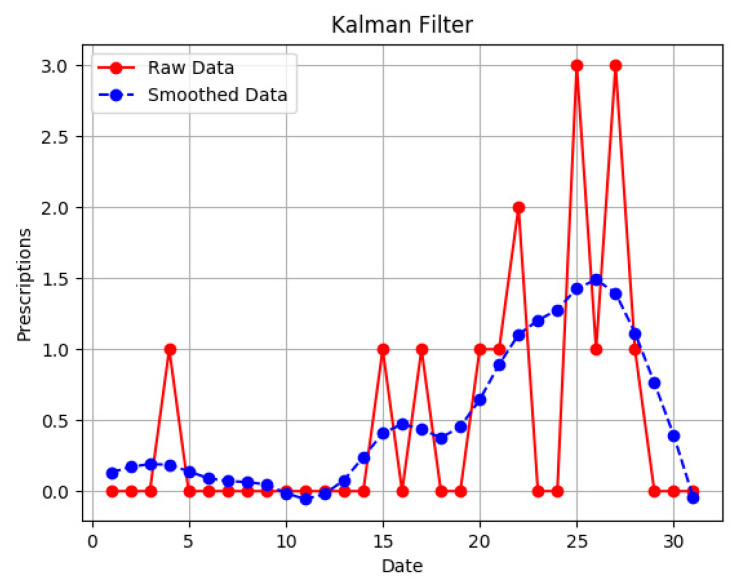
Comparison of raw data and smoothed data.

Plots of the data before and after smoothing with the Kalman smoother are shown in Figure A1.

#### 2.1.2. KDDI Location Data

The location data provided by KDDI were used for population flow estimation. GPS location data of all smartphones are collected 24 h a day, 365 days a year, and these data were extrapolated to match actual populations as closely as possible by combining them with sex/age-related information provided when users sign a mobile data contract and with public population statistics. A cell number was assigned to GPS data, and “stay” was determined when the logged data were present for more than 15 min in the same mesh. Otherwise, it was judged as “move”.

**Figure 1:**
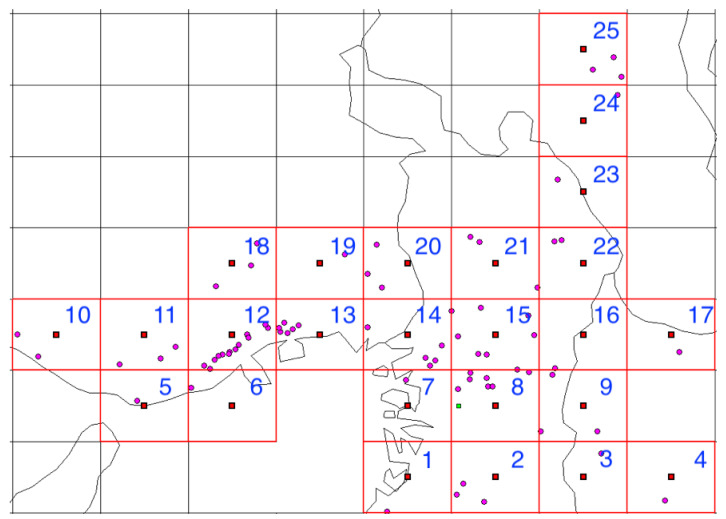
Cell number assignments for the analyzed region. Red grid lines: enclose the cells to be analyzed; Red solid squares: centers of the analyzed cells. Pink dots: pharmacies analyzed.

**Figure 2:**
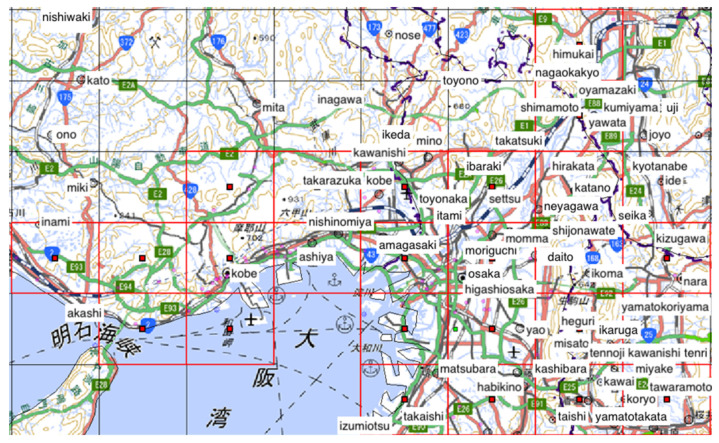
Map image of analyzed region. Red grid lines: enclose the cells to be analyzed. Red solid squares: centers of the analyzed cells. (The figure does not include all analyzed cells).

The analyzed region comprised the 25 cell areas each measuring 10 × 10 km^2^ in Osaka, Kyoto, Nara, and Hyogo prefectures shown in Figure 1 and Figure 2, in the month-long period from 1–31 December 2018.

#### 2.1.3. Population Flow Analysis Using Proposed Method Based on Neural Collective Graphical Models 

Neural collective graphical models (NCGMs) [17] constitute a fast and accurate method for estimating population flow [18], whereas collective graphical models (CGMs) [19] do not consider spatiotemporal dependencies. When estimating population flow Z, if the number of time points is defined as *T*, the number of areas as *L*, and the number of areas to which people can flow from a given cell as *M*, then the estimated parameter number is (T−1)LM, whereas the observed population statistics data are only *TL*, smaller than the estimated parameter number. It was with this last fact in mind that Iwata and Shimizu [17] proposed an NCGM, which uses a neural network to incorporate the spatiotemporal dependency issue and reduce the estimated parameters. The probability of a flow from a cell l to another cell $l^{\prime }$ at a time point t, defined as $\theta_{{tll}^{\prime }}$, is expressed as shown in Equation (6):

(6) $$\;\;\;\;\;\;\theta_{{tll}^{\prime }}=f\left(t,x_{l},x_{l^{\prime }};\varphi \right)\;\;\;\;\;\;$$

**Figure 3:**
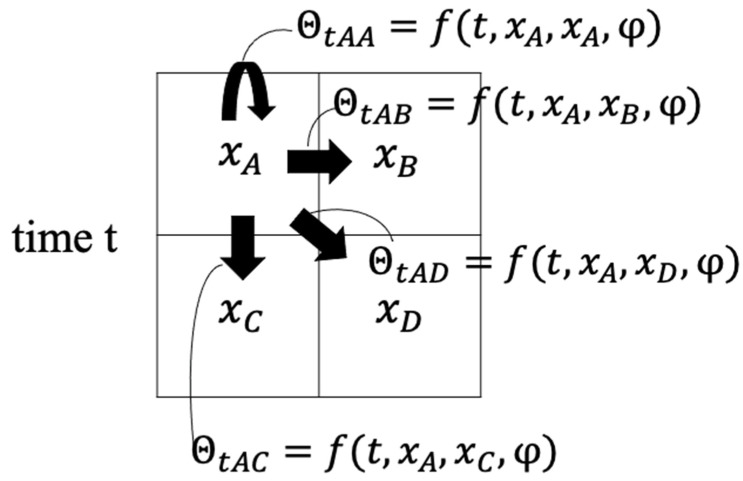
Model in which the number of areas is four.

Here, f(•) is the neural network, $x_{l}$ is the coordinate of the source cell, $x_{l^{\prime }}$ is the coordinate of the destination cell, and φ is a neural network parameter. This model outputs similar values for probabilities of transitions to areas at similar times and in similar directions. Figure 3 is a model for when there are 4 areas, labeled A, B, C, and D.

Each cell has information regarding the population and relative coordinates at a given time t. Using the flow from area A to area B as an example, the transition probability $\theta_{tAB}$ at time *t* was calculated in a nonlinear function constructed by the neural network (Equation (6)), with inputs of time *t*, source cell coordinates $x_{A},$ destination cell coordinates $x_{B},$ and neural network parameter φ.

The neural network input is expressed as the following vector:

(7) $$\;u_{{tll}^{\prime }}=\left[\tilde{\tau }\left(t\right),\tilde{x_{l}},\tilde{x_{l^{\prime }}}-\tilde{x_{l}}\right],$$

where *τ*(*t*) is the hour at time *t*, $\tilde{x}$ is x normalized between values from −0.5 to 0.5, and $u_{{tll}^{\prime }}\in R^{5}$ since the cell coordinates are two-dimensional. Next, this input vector was used in a three-layer feedforward neural network that applies the following activation functions to calculate the transition probabilities.

(8) $$h_{{tll}^{\prime }}=\tanh\left(W_{1}u_{{tll}^{\prime }}+b_{1}\right)$$

(9) $$\theta_{{tll}^{\prime }}=softmax\left(w_{2}h_{{tll}^{\prime }}+b_{2}\right)$$

Here, $h_{{tll}^{\prime }}\in R^{H}$ is the middle layer, where the number of units is H; and $W_{1}\in R^{H*5},\;w_{2}\in R^{H},\;b_{1}\in R^{H},\;\mathrm{and}\;b_{2}\in R$ are the estimated neural network parameters, or in other words, $\varphi =\left\{W_{1},w_{2},b_{1},b_{2}\right\}$.

(10) $$P\left(z_{tl}|\theta_{tl},y_{tl}\right)=\frac{y_{tl}!}{\prod_{l^{\prime }\in N_{l}}z_{{tll}^{\prime }}}\underset{l^{\prime }\in N_{l}}{\prod }\theta_{{tll}^{\prime }}^{z_{{tll}^{\prime }}}$$

(11) $$y_{tl}=\underset{l^{\prime }\in N_{l}}{\sum }z_{{tll}^{\prime }}$$

(12) $$y_{t+1,l}=\underset{l^{\prime }\in n_{l}}{\sum }z_{tl^{\prime }l}$$

The values of population flow *Z* and neural network parameter φ were estimated using maximum likelihood methods from Equations (10)–(12). The log-likelihood function L(Z,φ) is expressed as follows by taking the logarithm of both sides of Equation (10). Here, $z_{tll^{\prime }}$ and $y_{tl}$ are parameters for the population flow from a given cell *l* to another cell *l’* at a given time *t*, and the number of individuals in a given cell *l*, respectively.

(13) $$\begin{array}{c} L\left(Z,\varphi \right)=\overset{T-1}{\underset{t=1}{\sum }}\overset{L}{\underset{l=1}{\sum }}\underset{l^{\prime }\in N_{l}}{\sum }\log p(z_{tl}|\theta_{tl},y_{tl})\\ \propto \overset{T-1}{\underset{t=1}{\sum }}\overset{L}{\underset{l=1}{\sum }}\underset{l^{\prime }\in N_{l}}{\sum }-\log z_{tll^{\prime }}!+z_{tll^{\prime }}\log f\left(t,x_{l},x_{l^{\prime }};\varphi \right)\;\\ \approx \overset{T-1}{\underset{t=1}{\sum }}\overset{L}{\underset{l=1}{\sum }}\underset{l^{\prime }\in N_{l}}{\sum }z_{tll^{\prime }}(1-\log z_{tll^{\prime }}+\log f\left(t,x_{l},x_{l^{\prime }};\varphi \right))\;\\ \equiv L^{\prime }\left(Z,\varphi \right) \end{array}$$

Stirling’s approximation of logn!≈nlogn−n was used for the expression transformation from row 2 to 3 in Equation (13). Here, the objective function *G(z,φ)* to be optimized is expressed in the following equation when the constraint expressing the population conservation law was added as penalty terms in Equation (13).

(14) $$\begin{array}{c} G\left(Z,\varphi \right)=L^{\prime }\left(Z,\varphi \right)-\lambda_{1}\overset{T-1}{\underset{t=1}{\sum }}\overset{L}{\underset{l=1}{\sum }}||y_{tl}-\underset{l^{\prime }\in N_{t}}{\sum }z_{tll^{\prime }}|{\;|}^{2}\\ -\lambda_{2}\overset{T-1}{\underset{t=1}{\sum }}\overset{L}{\underset{l=1}{\sum }}||y_{t+1,l}-\underset{l^{\prime }\in N_{t}}{\sum }z_{tl^{\prime }l}|{\;|}^{2}\; \end{array}$$

The optimizations of *θ* and *φ* were determined with the gradient descent method after the transition probability *θ* was determined with a neural network. $\lambda_{1}>0$ and $\lambda_{2}>0$ are hyperparameters that control penalty terms. A summary of this estimation algorithm is shown in Appendix B as “Algorithm A1”.

## 3. Results

### 3.1. Estimation of Population Flow during Commuting Times

Murayama et al. [20] showed the effectiveness of using commuter data in epidemic prediction models and noted that models using commuter data, thought to be a factor in population flow, would have better results than models that use adjacent data between prefectures as spatial information. Furthermore, according to the 2016 Survey on Time Use and Leisure Activities [21] announced by the Statistics Bureau of the Japanese Ministry of Internal Affairs and Communications, the timeframe during which the percentage of moving persons (including commuters) peaks during weekdays is 8–9 am. Therefore, this research estimated population flows in the subject region in December 2018 during 8–9 a.m.

**Figure 4:**
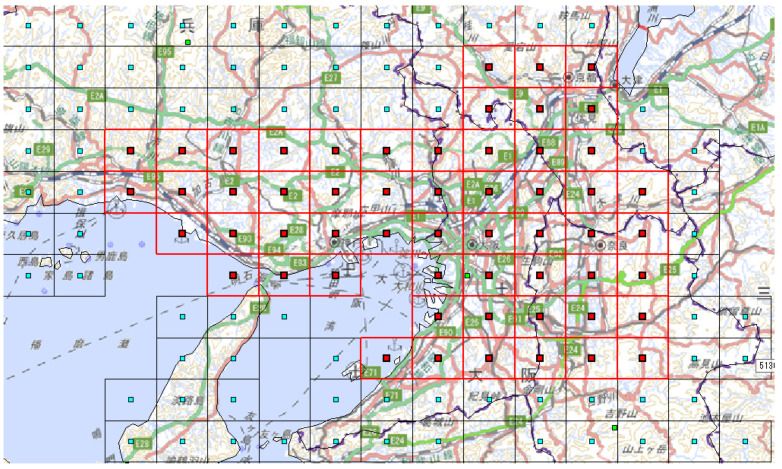
Region comprising the 25 analyzed cells and their neighboring areas that are connected by land. Red grid lines: enclose the cells to be analyzed. Red solid squares: centers of the analyzed cells. Black grid lines: areas excluded from analysis. Blue solid squares: centers of the unanalyzed cells.

**Figure 5:**
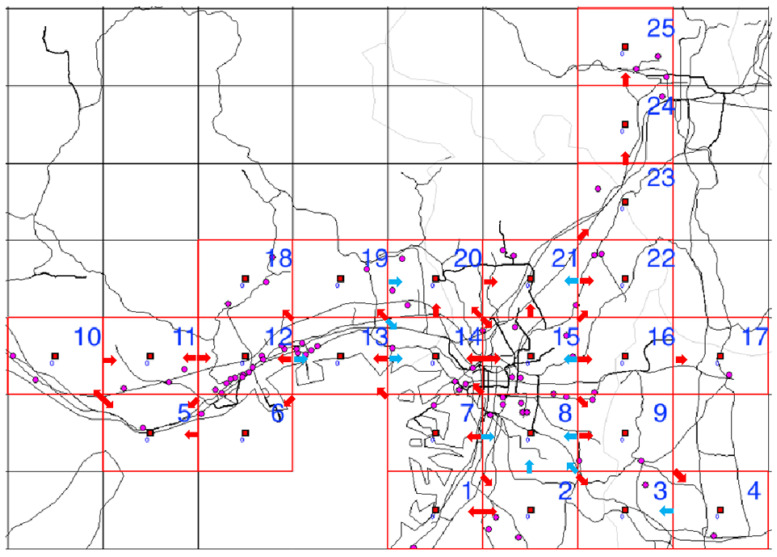
Population flow in the Kansai region during 8–9 a.m. Cells numbers: Arbitrarily assigned for analysis. Red grid lines: enclose the cells to be analyzed. Red solid squares: centers of the analyzed cells. Pink dots: pharmacies analyzed. Red arrows: indicate pathways with an average of over 50,000 moving persons on weekdays. Blue arrows: indicate pathways with an average of over 50,000 moving persons on weekends.

Methods proposed by Iwata and Shimizu (2019) and discussed in Section 2 were applied for all 25 cells to estimate population flow. The cells to which populations can move were designated as only the adjacent cells (including the original cell), and the initial values of population flow were weighted using the percentage of moving people during 8–9 a.m. in Table 2 (1)–(3). The circumscribing cells in addition to the 25 cells in the subject region were used when optimizing for population flow to improve estimation accuracy (Figure 4). Specific estimation results are shown later on, but the results of these procedures are shown in Figure 5, with red arrows indicating pathways with an average of over 50,000 moving persons on weekdays and blue arrows indicating pathways with an average of over 50,000 moving persons on weekends (black lines, excluding the grid lines, are railroad networks).

Population flow estimation results confirmed that there was high population flow in the east-west directions in cells 11, 12, and 13 along the Kobe coast and in the northern direction in cells 21, 23, and 24 from northeast Osaka towards Kyoto. A similarity between these pathways with high population flow is their traffic network development (primarily railroads). Furthermore, a characteristic point of population flow during the weekends is that it is directed towards Osaka. This is thought to be due to the fact that downtown areas and entertainment districts are concentrated within Osaka and that there are many people from outside the city who come for recreational purposes. Furthermore, there is relatively little population flow during 8–9 a.m. in cells 18 and 19. As can be seen in Figure 5, there are few railroads through cells 18 and 19, and this is thought to indicate how the extent of traffic network development plays a role in population flows during commuting times.

### 3.2. Relationships between the Estimates of Population Flow and the Number of Anti-Influenza Drug Prescriptions in Pharmacies

**Figure 6:**
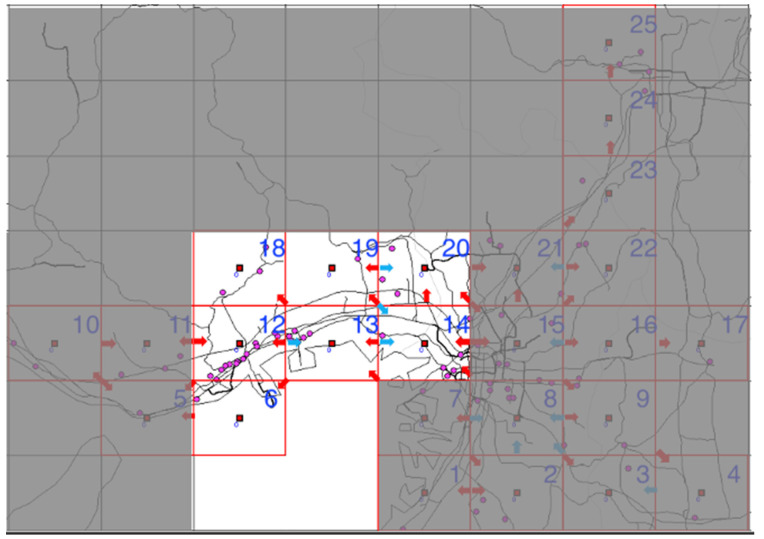
Population flow from cell 13 and its adjacent cells. Red grid lines: enclose the cells to be analyzed. Red solid squares: centers of the analyzed cells. Pink dots: pharmacies analyzed. Red arrows: indicate pathways with an average of over 50,000 moving persons on weekdays. Blue arrows: indicate pathways with an average of over 50,000 moving persons on weekends.

**Figure 7:**
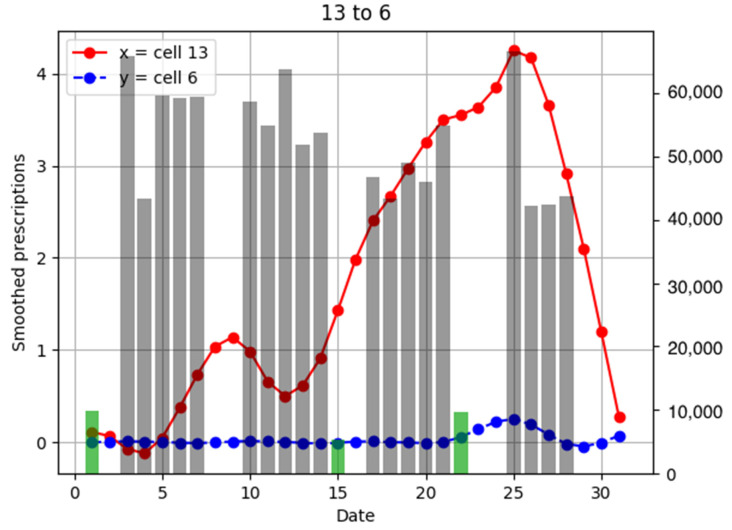
Population flow from cell 13 to cell 6 and the number of drug prescriptions in each cell. The y-axis on the right shows the population flow. Line graphs: number of smoothed prescriptions in cell. Gray bars: population flow on weekdays. Green bars: population flow on holidays.

**Figure 8:**
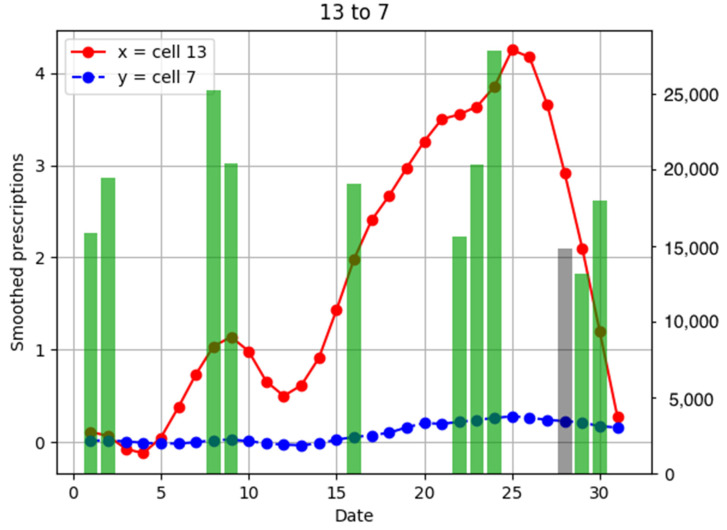
Population flow from cell 13 to cell 7 and the number of drug prescriptions in each cell. The y-axis on the right shows the population flow. Line graphs: number of smoothed prescriptions in cell. Gray bars: population flow on weekdays. Green bars: population flow on holidays.

**Figure 9:**
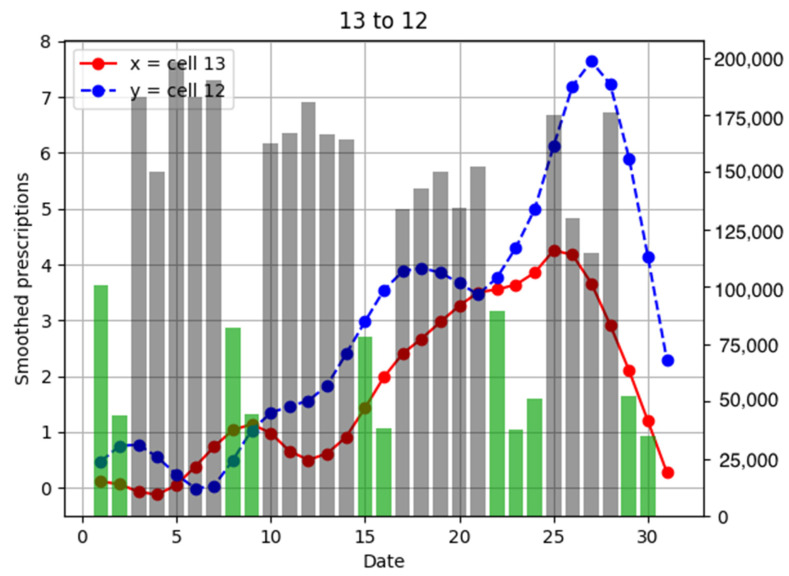
Population flow from cell 13 to cell 12 and the number of drug prescriptions in each cell. The y-axis on the right shows the population flow. Line graphs: number of smoothed prescriptions in cell. Gray bars: population flow on weekdays. Green bars: population flow on holidays.

**Figure 10:**
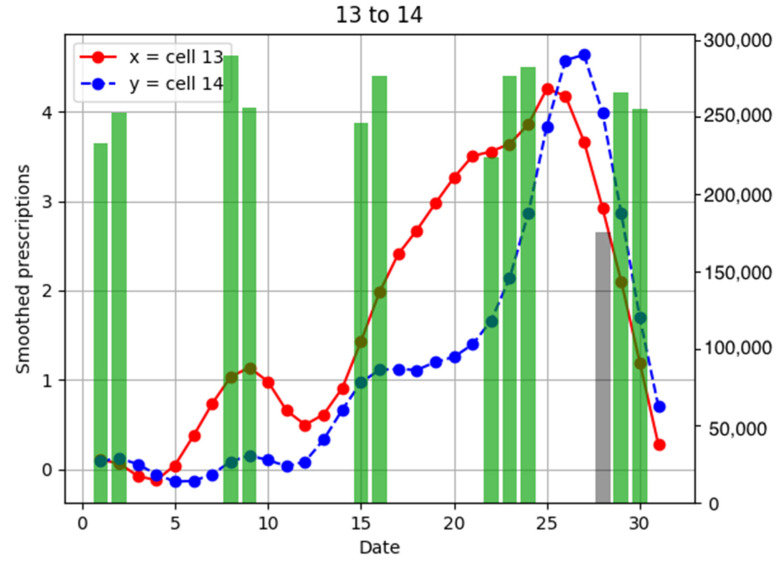
Population flow from cell 13 to cell 14 and the number of drug prescriptions in each cell. The y-axis on the right shows the population flow. Line graphs: number of smoothed prescriptions in cell. Gray bars: population flow on weekdays. Green bars: population flow on holidays.

**Figure 11:**
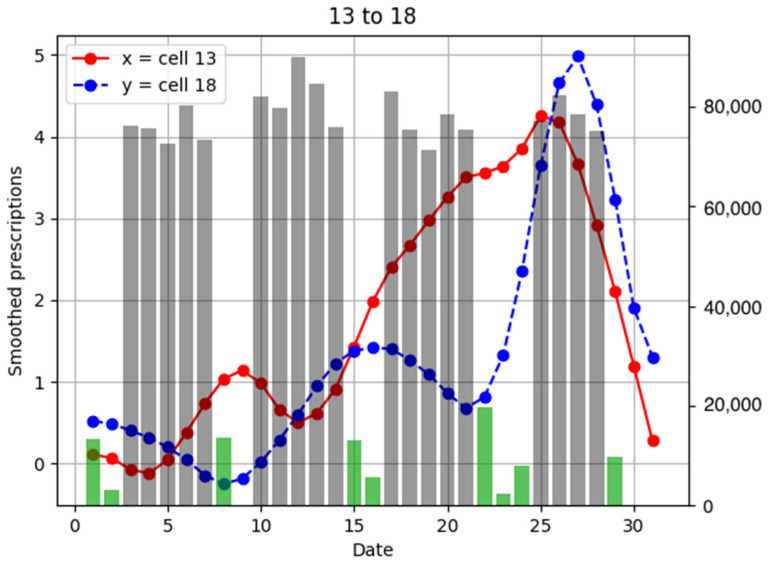
Population flow from cell 13 to cell 18 and the number of drug prescriptions in each cell. The y-axis on the right shows the population flow. Line graphs: number of smoothed prescriptions in cell. Gray bars: population flow on weekdays. Green bars: population flow on holidays.

**Figure 12:**
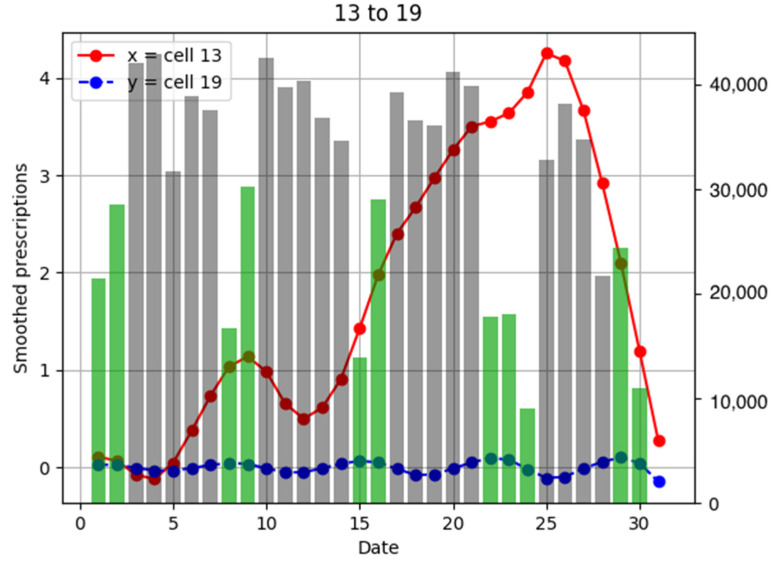
Population flow from cell 13 to cell 19 and the number of drug prescriptions in each cell. The y-axis on the right shows the population flow. Line graphs: number of smoothed prescriptions in cell. Gray bars: population flow on weekdays. Green bars: population flow on holidays.

**Figure 13:**
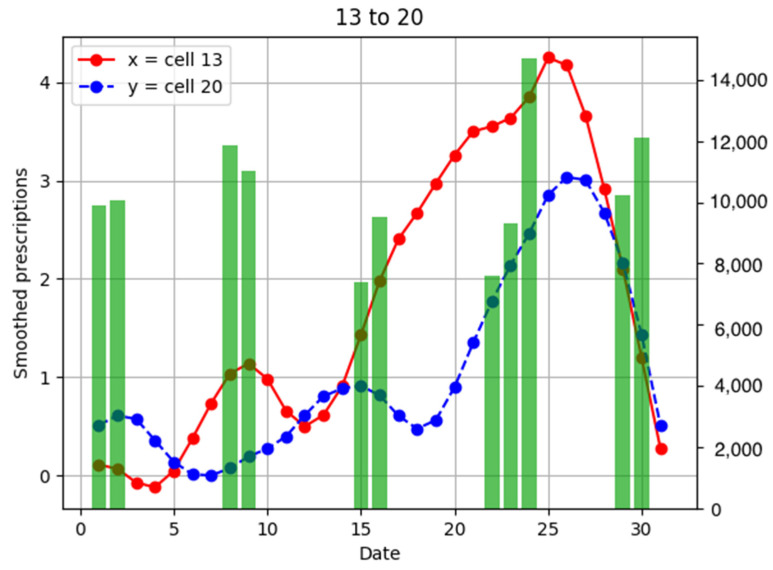
Population flow from cell 13 to cell 20 and the number of drug prescriptions in each cell. The y-axis on the right shows the population flow. Line graphs: number of smoothed prescriptions in cell. Gray bars: population flow on weekdays. Green bars: population flow on holidays.

To investigate the relationship between the number of anti-influenza drug prescriptions in pharmacies and population flows, examples of population flow between two cells through which individuals can move and the number of drug prescriptions from pharmacies in each cell are shown as graphs, focusing on areas with a large number of drug prescriptions (Figure 6, Figure 7, Figure 8, Figure 9, Figure 10, Figure 11, Figure 12 and Figure 13).

Furthermore, the cross-correlation functions of the number of drug prescriptions between two cells are shown as needed. The correlation between the number of drug prescriptions between geographically distant pharmacies can be expressed using the cross-correlation function to indicate the region-based temporal lag in the estimation of increases and decreases in the number of drug prescriptions. A shift has either a positive or a negative direction, and this acts as the horizontal axis of the cross-correlation function. The shift extent (number of days) is expressed as t.

Population flow and the number of drug prescriptions in cell 13 (Nishinomiya city, Hyogo Prefecture) and its surrounding areas are discussed first (Figure 6).

### 3.3. Population Flow from Cell 13 to Its Surroundings and the Number of Drug Prescriptions in Their Cells

Population flow from cell 13 was primarily on the weekdays towards cells 12 and 18 in Figure 7, Figure 8, Figure 9, Figure 10, Figure 11, Figure 12 and Figure 13. Line graphs describe the number of smoothed prescriptions in each cell. Gray bars show people who moved on weekdays, and green bars show people who moved on holidays in Figure 7, Figure 8, Figure 9, Figure 10, Figure 11, Figure 12 and Figure 13.

**Figure 14:**
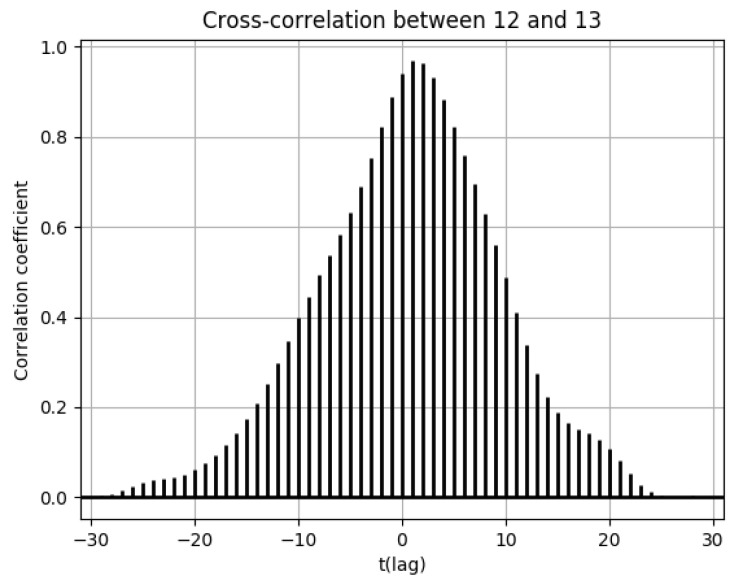
Cross-correlation function of the numbers of drug prescriptions between cells 12 and 13.

Cell 12 had the highest population flow from cell 13, which tended to be high during weekdays. When considering the distributions of the numbers of drug prescriptions of cells 12 and 13, population flows decreased immediately after the day 25–26 peak in cell 13, but considering the incubation period of the virus, we can see that the peak in cell 12 was 1–2 days later, on day 27 in Figure 7. This corresponds to the time lag of 1–2 of the cross-correlation functions of the numbers of drug prescriptions between cells 12 and 13 shown in Figure 14.

**Figure 15:**
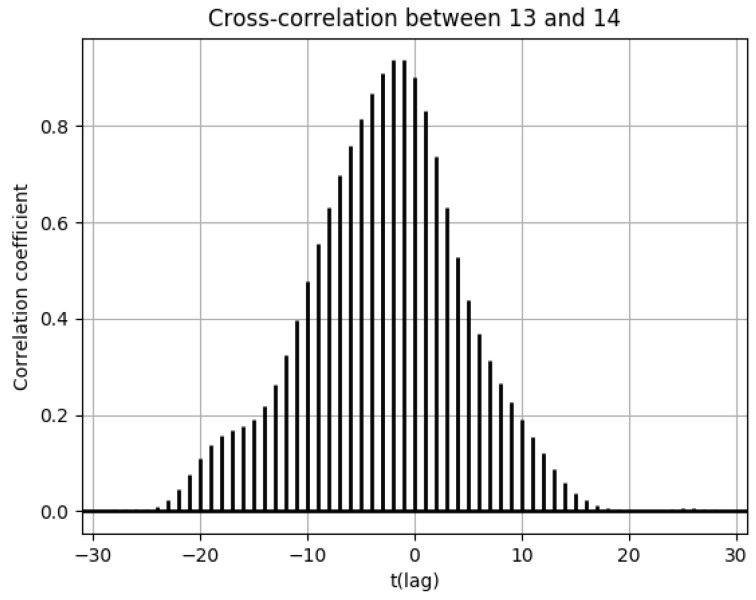
Cross-correlation function of the numbers of drug prescriptions between cells 13 and 14.

Population flows from cell 13 to cell 14 were high during the weekend, but the peak number of drug prescriptions in cell 14 came 2 days after the peak in cell 13. This corresponds to the time lag of −2 in the cross-correlation function of the numbers of drug prescriptions between cells 13 and 14 in Figure 15.

There was no increased number of drug prescriptions in cells 6, 19, 20, and 7, which had low population flows.

### 3.4. Population Flows from Cell 6 and Its Surroundings and the Number of Drug Prescriptions in Their Cells

**Figure A2:**
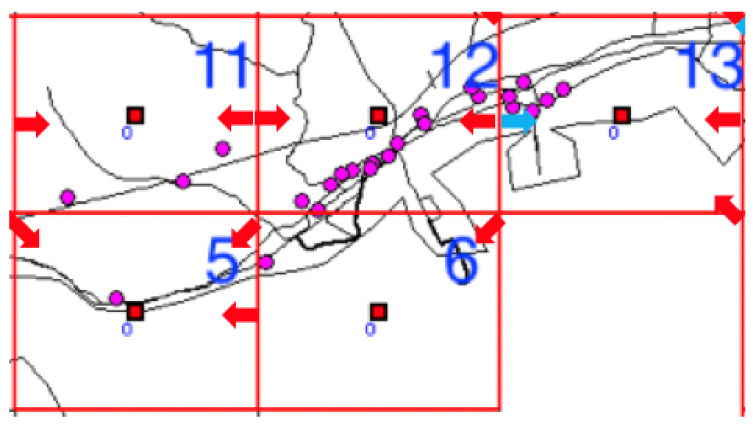
Population flow from cell 6 and its adjacent cells.

In this section, population flow and the number of drug prescriptions in cell 6 (southeastern Kobe city, Hyogo Prefecture) and its surrounding areas are discussed (Figure A2).

Population flows from cell 6 were highest in the direction of cell 5 in Figure A3, Figure A4, Figure A5 and Figure A6. However, the numbers of drug prescriptions in both cells were low. The scale of each graph shown in Figure 7, Figure 8, Figure 9, Figure 10, Figure 11, Figure 12 and Figure 13, Figure A3, Figure A4, Figure A5 and Figure A6, and Figure A7, Figure A8, Figure A9, Figure A10 and Figure A11 and Figure A13, Figure A14, Figure A15, Figure A16, Figure A17, Figure A18, Figure A19 and Figure A20 shown later, depends on the individual graph. It is clear that there is still no major spread of influenza. However, although small, there is no major difference between the numbers of drug prescriptions in cell 5 and cell 6 at the peak time. Therefore, the possibility of infection spread from cell 5 to cell 6 is considered to be high. The cross-correlation functions in the numbers of drug prescriptions between the two cells are shown in Figure 16.

**Figure 16:**
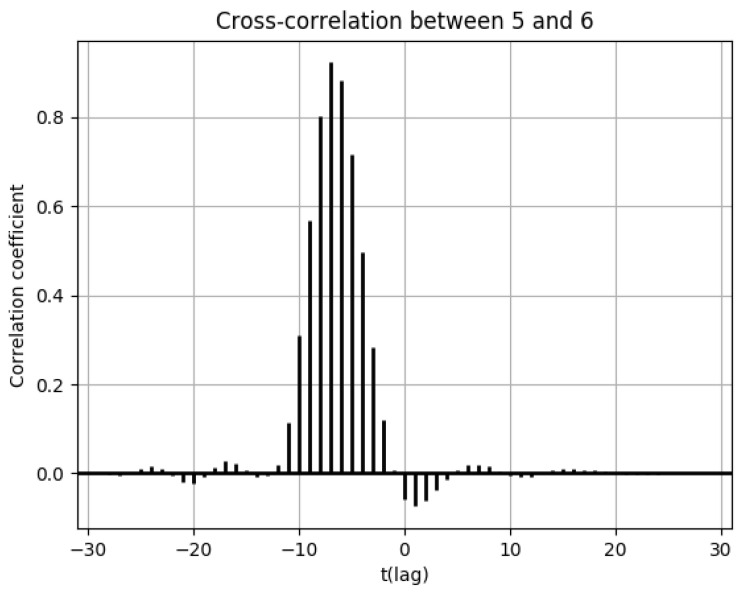
Cross-correlation function of the numbers of drug prescriptions between cells 5 and 6.

From the cross-correlation function in Figure 16, it is clear that cross-correlations were high at t = −7. In other words, infections spread along the pathway from cell 5 to cell 6, and a week-long lag was present for infection. Furthermore, population flow into cell 5 decreased once a peak of the number of drug prescriptions was reached in cell 6, so the spread of influenza was thought to affect fluctuations in population flow.

Furthermore, there was not much population flow from cell 6 to cells 11, 12, and 13, and the numbers of drug prescriptions were significantly lower in cells 11, 12, and 13 than in cell 6. It can thus be seen that influenza infection in cells 11, 12, and 13 had a low correlation with influenza infection in cell 6.

### 3.5. Population Flows from Cell 19 and Its Surroundings and the Numbers of Drug Prescriptions in Their Cells

In this section, population flow and the number of drug prescriptions in cell 19 (Nishinomiya city, Hyogo Prefecture) and its surrounding areas are discussed (Figure A12).

**Figure 17:**
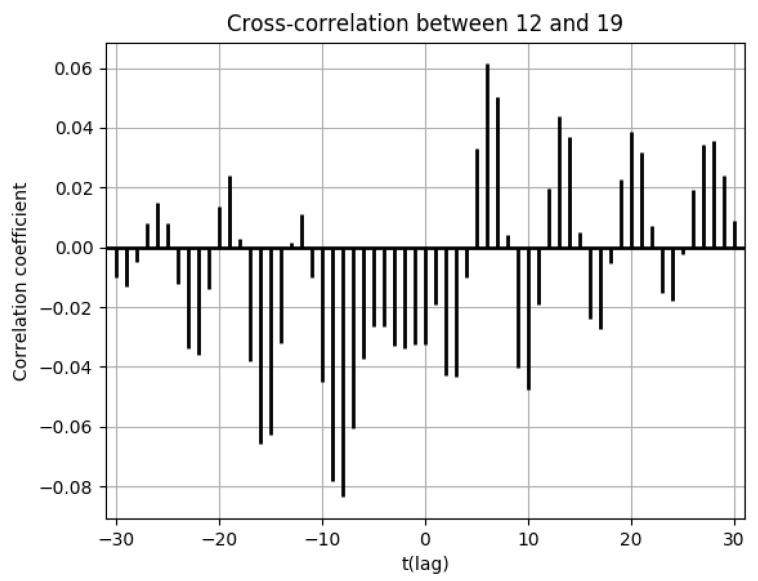
Cross-correlation function of the numbers of drug prescriptions between cells 12 and 19.

**Figure A7:**
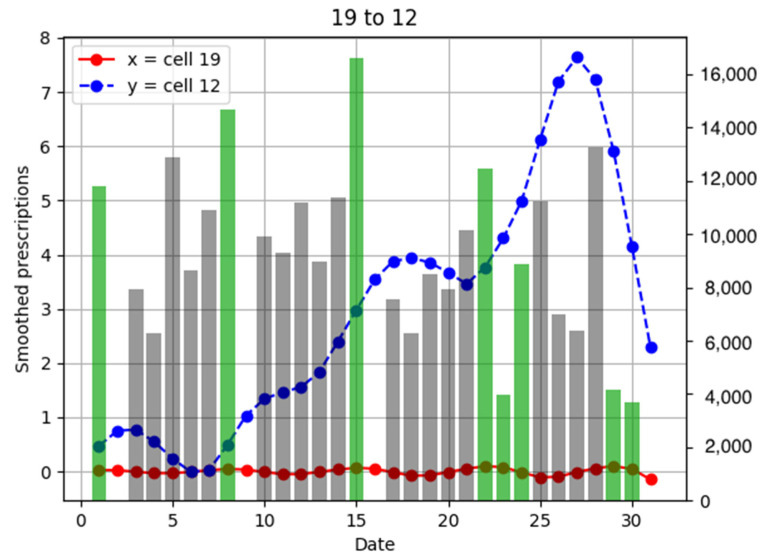
Population flow from cell 19 to cell 12 and number of drug prescriptions in each cell.

**Figure A8:**
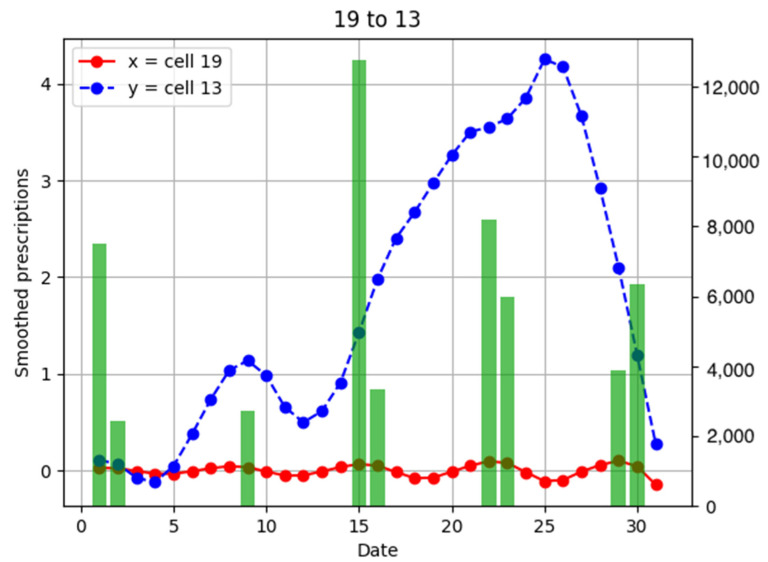
Population flow from cell 19 to cell 13 and the number of drug prescriptions in each cell.

**Figure A9:**
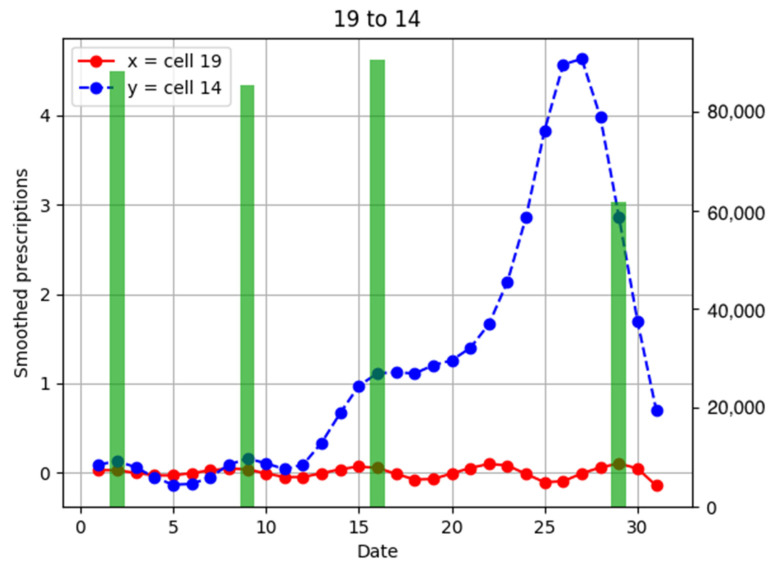
Population flow from cell 19 to cell 14 and number of drug prescriptions in each cell.

**Figure A10:**
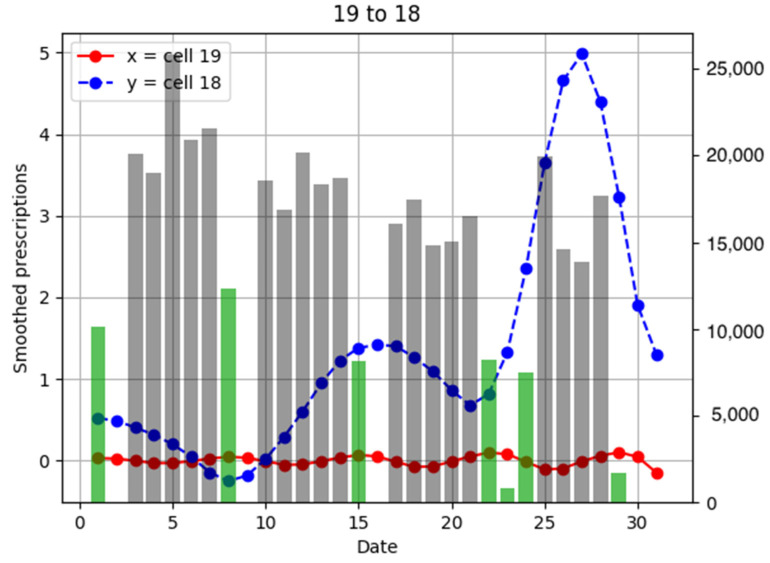
Population flow from cell 19 to cell 18 and number of drug prescriptions in each cell.

**Figure A11:**
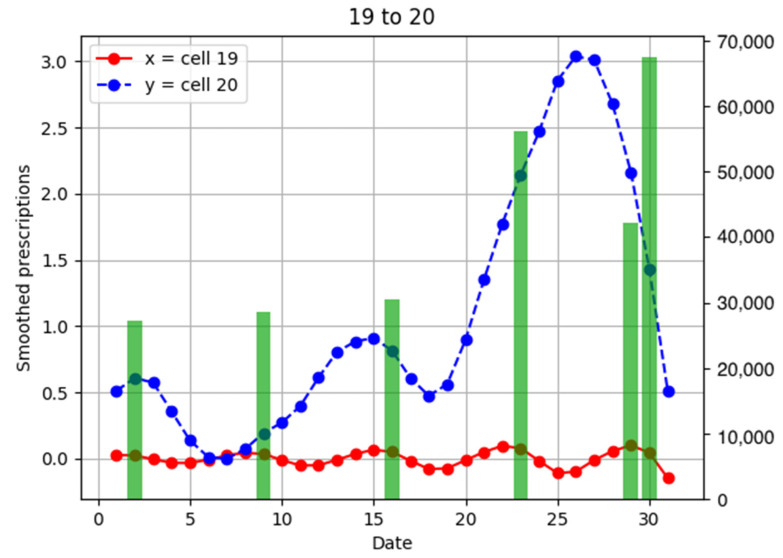
Population flow from cell 19 to cell 20 and number of drug prescriptions in each cell.

**Figure A12:**
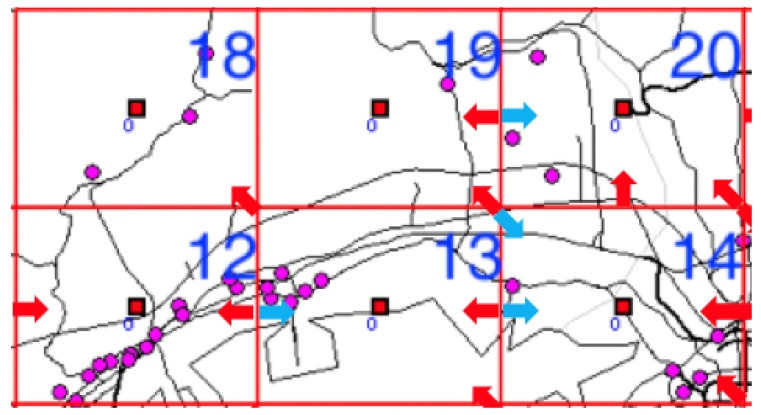
Population flow from cell 19 and its adjacent cells.

The low population flows to external areas on the weekdays from cell 19 were attributed to undeveloped traffic networks (Figure A7, Figure A8, Figure A9, Figure A10 and Figure A11). For this reason, the number of drug prescriptions in cell 19 was also small, and it can be determined from the cross-correlation functions that there was virtually no external spread of infection (Figure 17).

### 3.6. Population Flows from Cell 8 and Its Surroundings and the Number of Drug Prescriptions in Their Cells

**Figure 18:**
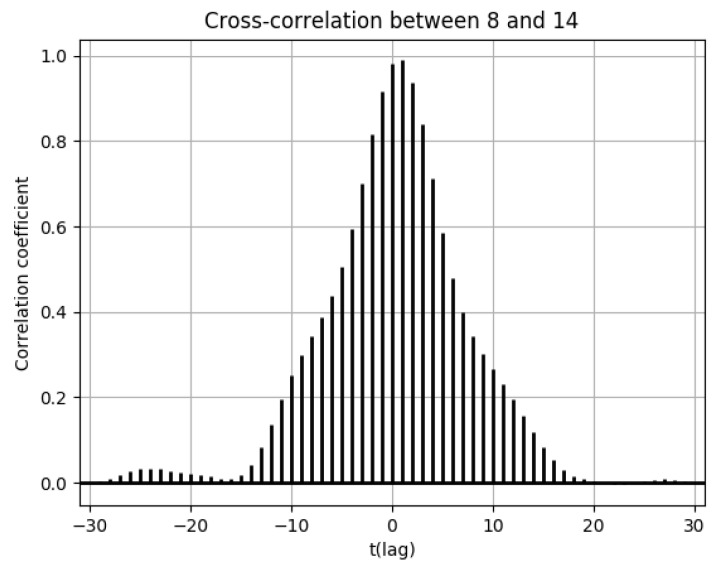
Cross-correlation function of the numbers of drug prescriptions between cells 8 and 14.

**Figure 19:**
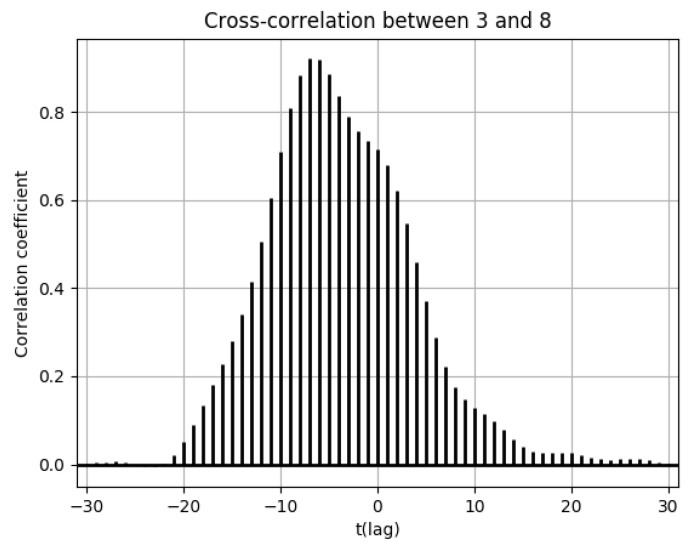
Cross-correlation function of the numbers of drug prescriptions between cells 3 and 8.

**Figure A13:**
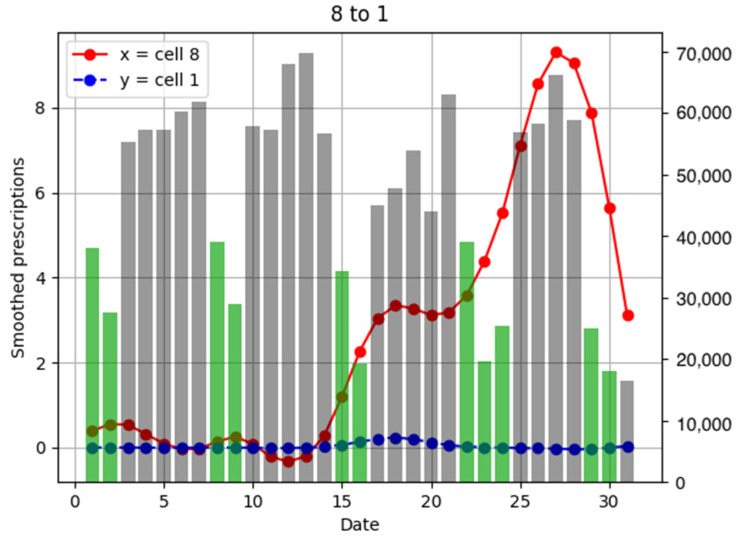
Population flow from cell 8 to cell 1 and number of drug prescriptions in each cell.

**Figure A14:**
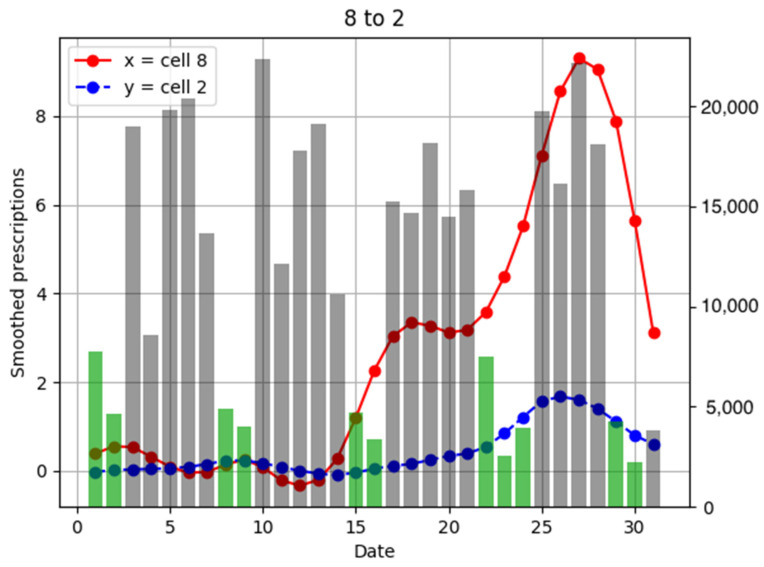
Population flow from cell 8 to cell 2 and number of drug prescriptions in each cell.

**Figure A15:**
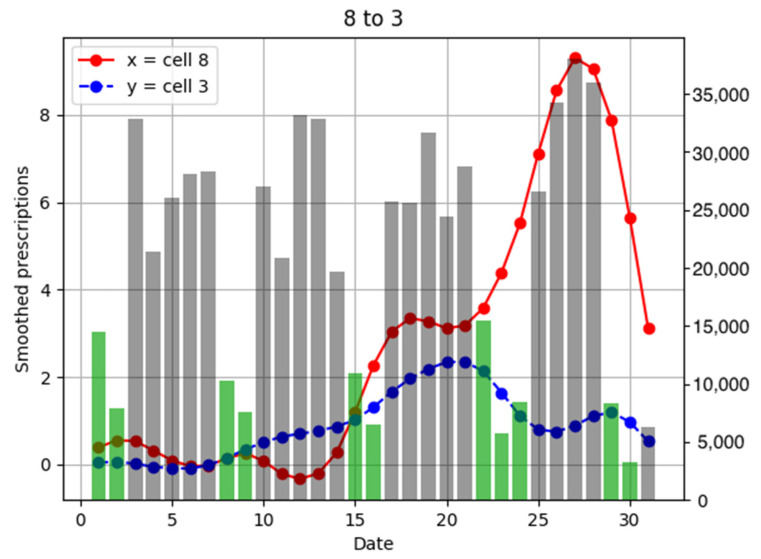
Population flow from cell 8 to cell 3 and number of drug prescriptions in each cell.

**Figure A16:**
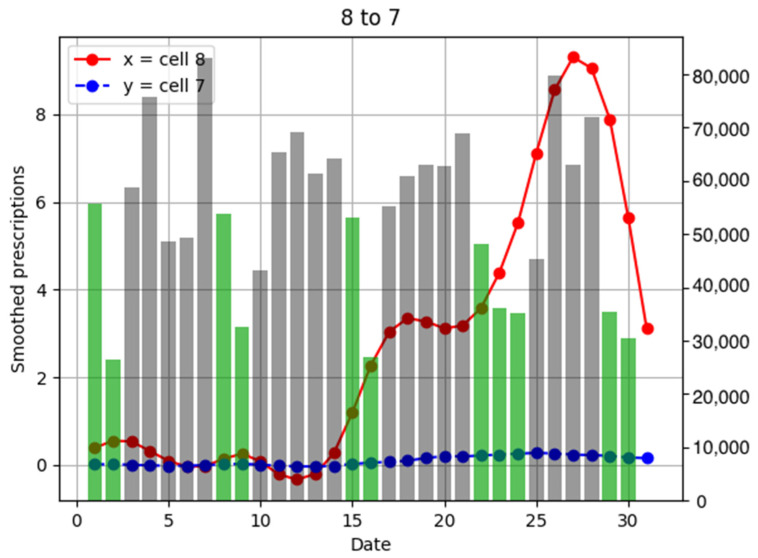
Population flow from cell 8 to cell 7 and number of drug prescriptions in each cell.

**Figure A17:**
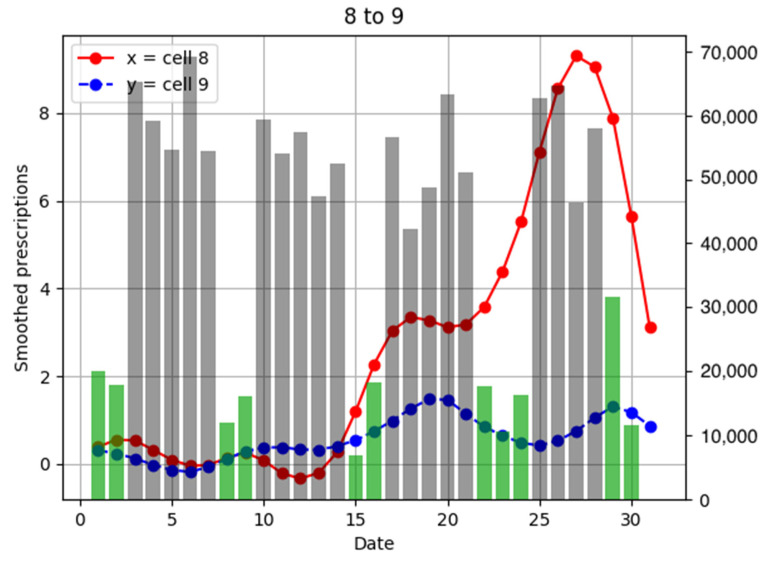
Population flow from cell 8 to cell 9 and number of drug prescriptions in each cell.

**Figure A18:**
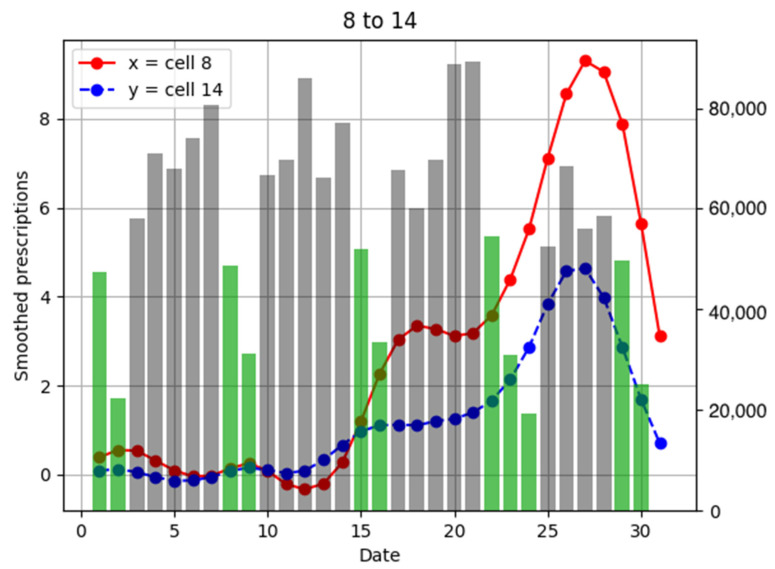
Population flow from cell 8 to cell 14 and number of drug prescriptions in each cell.

**Figure A19:**
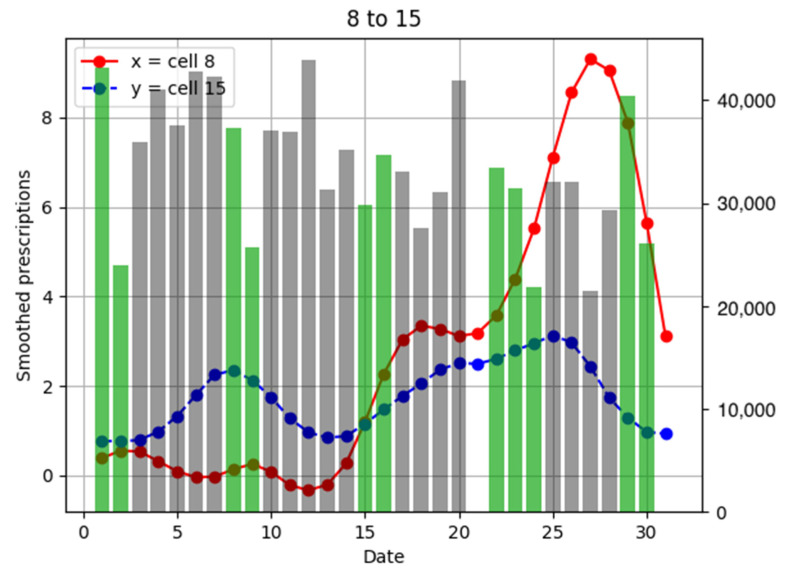
Population flow from cell 8 to cell 15 and number of drug prescriptions in each cell.

**Figure A20:**
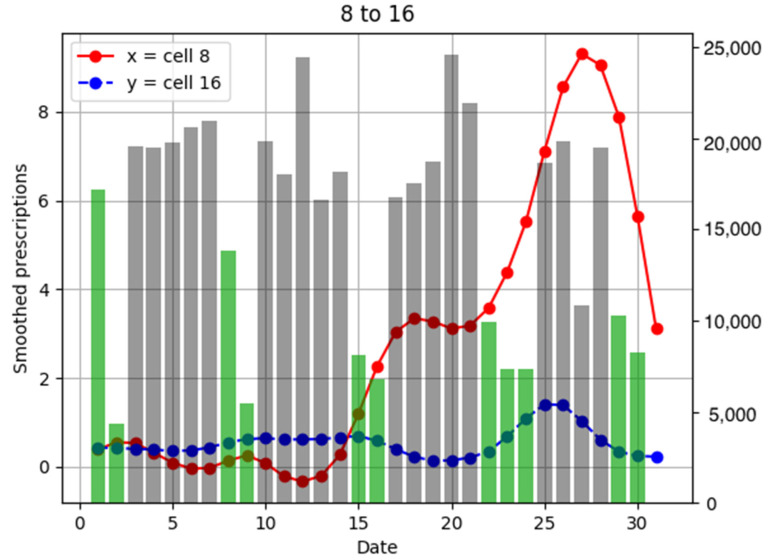
Population flow from cell 8 to cell 16 and number of drug prescriptions in each cell.

**Figure A21:**
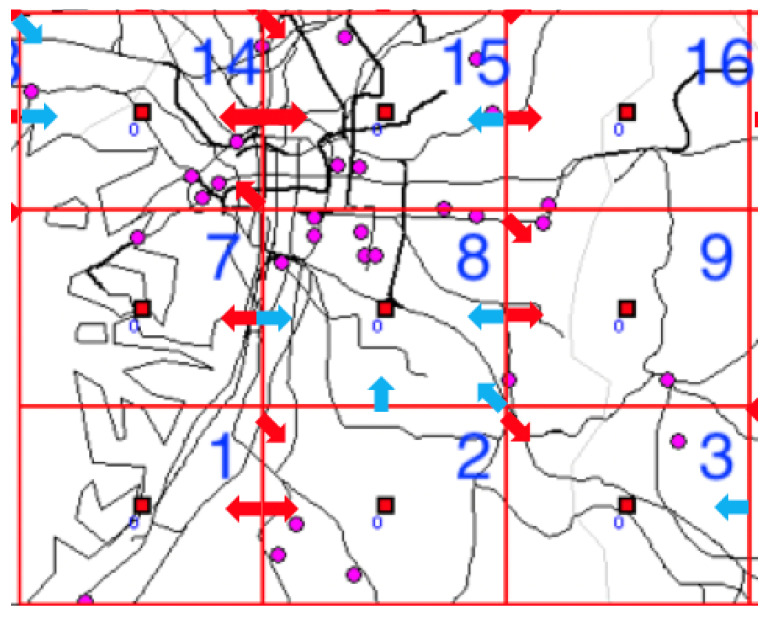
Population flow from cell 8 and its adjacent cells.

In this section, population flow and the number of drug prescriptions in cell 8 (Osaka city, Osaka Prefecture) and its surrounding areas are discussed (Figure A21). Population flow from cell 8 was highest towards cell 3, followed by cell 14 (Figure A13, Figure A14, Figure A15, Figure A16, Figure A17, Figure A18, Figure A19 and Figure A20). However, when comparing (3) and (6), it seemed that the distribution of the number of drug prescriptions in cell 8 had a higher correlation with cell 14 than cell 3. This was because the number of drug prescriptions was higher in cell 14 than in cell 3, and the pharmacy locations in cell 8 were closer to cell 14. For this reason, it can be seen in Figure 18 that there was virtually no lag in the correlation of the numbers of drug prescriptions between cells 8 and 14. Meanwhile, Figure 19 shows that there was a time lag of −7 for the correlation of the numbers of drug prescriptions between cells 3 and 8.

## 4. Discussion

### 4.1. Principal Results and Interpretation

#### 4.1.1. Observing the Population Flow from Cell 13 to Its Surroundings and the Number of Prescriptions in Those Cells

The prescription peaks in cells 12 and 14, which had high population flows with cell 13, showed a high correlation with a delay of 1–2 days. The time from infection to onset, the incubation period, is 1–4 days (average 2 days) in seasonal influenza [22,23]. The delayed peak suggests the spread of influenza from cell 13 to the surrounding areas.

#### 4.1.2. Observing the Population Flow from Cell 6 to Its Surroundings and the Number of Prescriptions in Those Cells

Two observations in this region are noteworthy. One of the features around cell 6 is the low number of prescriptions for anti-influenza drugs. We suspect that the influenza infection may not have spread to cell 6 due to the low population flow from cells 12 and 13 with high prescriptions. Another feature is the observation of transmission of infection by a small number of influenza patients. In cells 5 and 6, where high population flows were suspected, there was a high cross-correlation value of prescription numbers with a 7-day time-lag. We believe this phenomenon indicates the spread of influenza infection from cell 6 to cell 5. The observed 7-day time lag is longer than the time lag observed around cell 13 above. There may be a difference in the speed of spread between when there is and is not an influenza outbreak.

#### 4.1.3. Observing the Population Flow from Cell 19 to Its Surroundings and the Number of Prescriptions in Those Cells

It was observed that not much population flows from cell 19 to the surrounding areas on weekdays. This observation may have been due to geographical features and the undeveloped transportation networks. The number of prescriptions for anti-influenza drugs in cell 19 remained low during the observation period. The small population inflow from the surrounding areas may have been a deterrent to the influenza epidemic.

#### 4.1.4. Observing the Population Flow from Cell 8 to Its Surroundings and the Number of Prescriptions in Those Cells

The number of prescriptions for anti-influenza drugs at closely located pharmacies showed a high correlation with no time difference. On the other hand, the correlation of the number of prescriptions at distant pharmacies showed a time difference, even in a cell with high population flows. The number of anti-influenza drug prescriptions around cell 8 appeared to be affected by distance and the number of pharmacies. A more detailed estimate of population flows in the cell may be useful for explaining the time difference shown.

### 4.2. Principal Findings

Relationships between population flows and prescriptions for anti-influenza drugs were evaluated in multiple areas. We visualized the relationship between population flow and influenza infection epidemics on a single graph with the passage of time, and mathematically evaluated the relationship of the number of anti-influenza prescriptions between neighboring areas using the time lag by the cross-correlation function. The two types of information helped to estimate the direction and volume of population flow and the direction and rate of spread of influenza infection. Observations of influenza pandemics based on the population flow, between 10 km square areas, from 8:00 to 9:00, taking into account the day of the week, provide interesting implications. Our results support the notion that transportation networks such as trains play an important role in the spread of the influenza pandemic. This is because the time when the population flow was observed is the commuting time, and railroads are laid as a transportation network in areas with abundant flow of people. It was found that influenza did not spread to areas with undeveloped traffic networks, and the peak number of drug prescriptions arrived with a time lag of several days in areas where there was a high amount of area-to-area movement due to commuting. Our results may also provide additional suggestions. If there are few influenza patients, the rate of transmission may be slow even between areas with high population flow. Even between neighboring 10 km square areas, the spread of influenza infection is affected by distance and may take some time to spread.

### 4.3. Limitations

The results of the present study should be considered in light of several limitations. First, only about 1% of the pharmacies in the Kansai region could be observed. Each of the observed pharmacies varied in scale, and they were unevenly distributed within the cells. Inhomogeneous information from a limited number of pharmacies will adversely affect predictions. Therefore, it is necessary to increase the number of pharmacies to allow more accurate predictions to be made.

Second, anti-influenza drug prescriptions were used as a surrogate measure of influenza infections. Although the prescription status of anti-influenza drugs is considered to indicate the epidemic status of influenza [8,9], not all influenza patients can be traced, because some influenza patients are not prescribed anti-influenza drugs. Therefore, electronic medical record information should be analyzed for more accurate influenza epidemic tracking.

Third, means of transportation were not investigated. People move by various means such as cars, trains, buses, and walking. The impact of different modes of transportation on influenza infection is still unclear [5]. We believe it is necessary to clarify the relationships between the different forms of transport and the spread of influenza transmission.

Finally, while mobile phone location tracking technology has been suggested to be helpful for preventing the spread of communicable diseases, its efficacy remains controversial [24]. The questions of “who,” “how,” and “what” GPS data are to be used remain unanswered. We used anonymous aggregated location data to predict the population flow. Although the location data covered a large sample of the Japanese population, the selection can be biased because the data were provided by one company. Predicting real-time population flow was also not possible due to the time needed to provision.

### 4.4. Comparison with Prior Work

Many previous reports have attempted to explain the spread of influenza infection mathematically and track influenza epidemics based on internet social network service information [25,26,27,28,29]. To predict influenza-endemic areas, data from internet social network services were commonly used in the previous studies. In the present study, the population flow information that is not directly related to influenza was analyzed, and it was compared to the influenza pandemic. There have recently been cases of using GPS to track several infectious diseases [9,10,11,30,31]. In these previous reports, authors made various attempts, such as using information from GPS to track the mobility of individual cases and assess trends in epidemics in the region. Information obtained from GPS can provide a new tool for combatting viral infectious diseases such as influenza, COVID-19, and HIV. The unique feature of the present study was to predict the population flow during commuting time and mathematically evaluate the number of anti-influenza prescriptions between relatively small neighboring areas with a cross-correlation function. An examination of the flow of people focused on a limited time period in a particular neighborhood showed that commuter trains may play an important role in the spread of influenza infection. The time lag given by the cross-correlation function of anti-influenza prescriptions in neighboring areas may provide information about the direction of spread of influenza infection. We believe that such information is important for the prevention and prediction of influenza pandemics in neighboring areas.

## 5. Conclusions

Population flows during commuting times were estimated based on location data, and a region-specific analysis of infection pathways was conducted by examining in detail the relationship between the estimated population flow and the number of influenza drug prescriptions. It was found that influenza did not spread to areas with undeveloped traffic networks, and the peak number of drug prescriptions arrived with a time lag of several days in areas with a high amount of area-to-area movement due to commuting. Estimated population flow based on region-specific GPS location data may have an important role in predicting the spread of influenza infection. However, this method still has room for improvement, including real-time prediction and the use of unbiased information sources. In order to overcome the limitations of this study, we would like to obtain a wide range of information and improve the algorithm and thereby contribute to the prediction and prevention of epidemics of infectious diseases such as influenza.

## Figures and Tables

**Table 1:** The number of prescriptions for each day of the week (for cases where Sunday is a closed day).

Day of the Week	The Number of Prescriptions
Friday	4
Saturday	3
Sunday	0
Monday	20
Tuesday	6
Wednesday	9
Thursday	7

**Table 2:** Percentage of moving persons by morning hour.

Timeframe	Percentage of Total (%)
(1) Weekdays	
5:00–6:00	1.475
6:00–7:00	6.275
7:00–8:00	15.828
8:00–9:00	15.870
9:00–10:00	9.858
10:00–11:00	10.268
(2) Saturday	
5:00–6:00	1.780
6:00–7:00	4.250
7:00–8:00	10.995
8:00–9:00	12.585
9:00–10:00	14.570
10:00–11:00	14.348
(3) Sunday	
5:00–6:00	1.343
6:00–7:00	2.843
7:00–8:00	5.638
8:00–9:00	11.118
9:00–10:00	15.648
10:00–11:00	16.113

**Figure A3:**
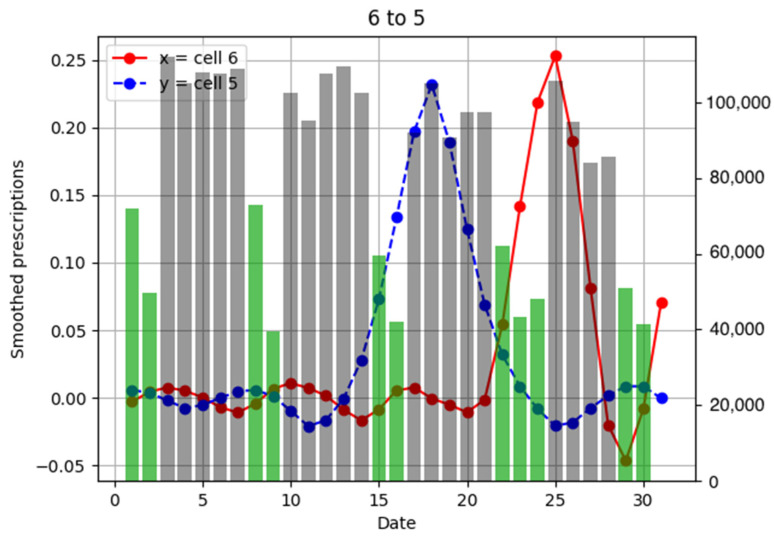
Population flow from cell 6 to cell 5 and number of drug prescriptions in each cell.

**Figure A4:**
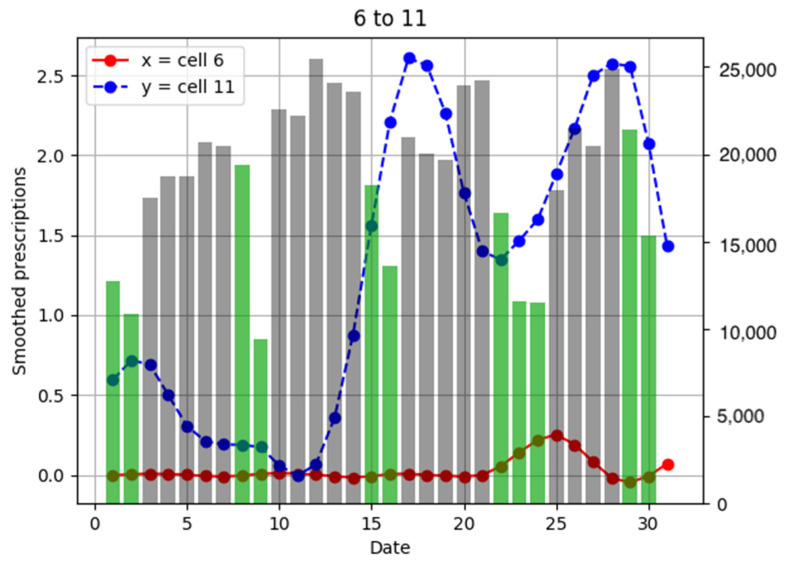
Population flow from cell 6 to cell 11 and number of drug prescriptions in each cell.

**Figure A5:**
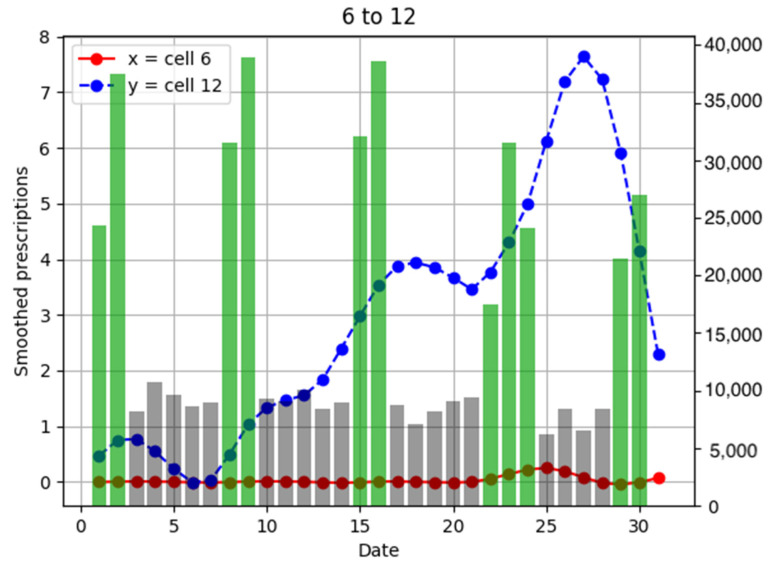
Population flow from cell 6 to cell 12 and number of drug prescriptions in each cell.

**Figure A6:**
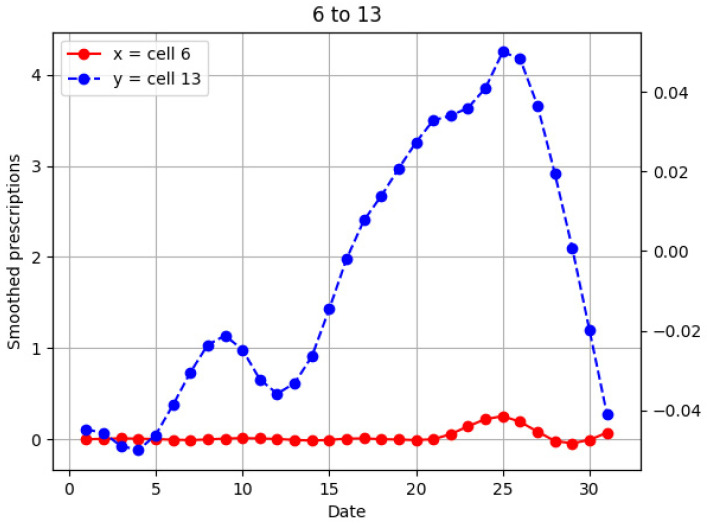
Population flow from cell 6 to cell 13 and the number of drug prescriptions in each cell. There was very little population flow between cells 6 and 13. Therefore, the Y-axis on the right shows provisional values created by the program.

## Data Availability

The datasets generated during and/or analyzed during the current study are not publicly available because of access to information being severely restricted by the ethics committee of Juntendo University, but they may be available from the corresponding author on reasonable request.

## Appendix B

Summary of Algorithm A1.

**Algorithm A1.** Estimation procedure for the proposed NCGM.	
**Require:**	Spatio-temporal population data Y location information ${\left\{x_{t}\right\}}_{t=1}^{L}\;$ neighbor information ${\left\{N_{l}\right\}}_{l=1}^{L}$ hyperparameter λ
**Ensure:**	Population flow Z estimated neural network parameters φ; **Repeat** Calculate transition probability $\theta_{tl}$ by neural network f for t=1 to T−1**,** l=1 to L; Calculate the objective function G and its gradient with respect to Z and φ; Update Z and φ using gradient; **until** End condition is satisfied
